# Mechanical Properties of Nonwoven Reinforced Thermoplastic Polyurethane Composites

**DOI:** 10.3390/ma10060618

**Published:** 2017-06-05

**Authors:** Muhammad Tausif, Achilles Pliakas, Tom O’Haire, Parikshit Goswami, Stephen J. Russell

**Affiliations:** Textile Technology Research Group, School of Design, University of Leeds, Leeds LS2 9JT, UK; A.Pliakas@leeds.ac.uk (A.P.); ohaire.tom@googlemail.com (T.O.); P.Goswami@leeds.ac.uk (P.G.); s.j.russell@leeds.ac.uk (S.J.R.)

**Keywords:** nonwovens, flexible, composites, fibre reinforced, mechanical properties, low cost

## Abstract

Reinforcement of flexible fibre reinforced plastic (FRP) composites with standard textile fibres is a potential low cost solution to less critical loading applications. The mechanical behaviour of FRPs based on mechanically bonded nonwoven preforms composed of either low or high modulus fibres in a thermoplastic polyurethane (TPU) matrix were compared following compression moulding. Nonwoven preform fibre compositions were selected from lyocell, polyethylene terephthalate (PET), polyamide (PA) as well as para-aramid fibres (polyphenylene terephthalamide; PPTA). Reinforcement with standard fibres manifold improved the tensile modulus and strength of the reinforced composites and the relationship between fibre, fabric and composite’s mechanical properties was studied. The linear density of fibres and the punch density, a key process variable used to consolidate the nonwoven preform, were varied to study the influence on resulting FRP mechanical properties. In summary, increasing the strength and degree of consolidation of nonwoven preforms did not translate to an increase in the strength of resulting fibre reinforced TPU-composites. The TPU composite strength was mainly dependent upon constituent fibre stress-strain behaviour and fibre segment orientation distribution.

## 1. Introduction

Composites combine two or more distinct materials with a discrete separating interface [1,2]. The two materials are typically heterogeneous but can consist of different physical forms of the same material [3]. In polymer-matrix composites, polymer resin forms the matrix of the composite and also acts as a binder and a stress transfer medium for the reinforcement component, which typically consists of fibres or textile structures [1,2,4,5]. The matrix can consist of an elastomeric, thermoset or thermoplastic polymer depending on whether a flexible or structural composite is required [6,7]. Elastomeric matrix polymers are capable of large deformations before failure compared to thermoset and thermoplastics making them particularly useful in the design of flexible composites. Flexible composite applications include hoses, tyres, coated fabrics, inflatables, conveyor belts, surgical devices, diaphragms and reinforced membranes [8,9].

Controlling the reaction of various polyols and isocyanates readily modifies the mechanical, chemical and thermal properties of polyurethanes [10,11,12]. In TPU, the hard segments are connected by polyether or polyester-based soft segments that provide the characteristic flexibility in the polymer and the segments are phase separated, resulting in hard and soft segment domains [13]. Thermoplastic polyurethanes (TPU) are versatile materials which can be processed into moulded parts [4], films [14], fibres [15] and reinforced composites [14]. TPUs exhibit good strength, excellent resistance to abrasion, extremely high strain to fracture, durability and high toughness [11,16]. The chemistry of the polymer as well as processing route allows it to attain a wide range of mechanical, thermal and performance properties. For example, the tensile strength/elongation at break of commercial grade Elastollan^®^ C 88 A 10 and 1278 D 11 U (BASF Polyurethanes GmbH, Ludwigshafen, Germany) is 50 MPa/600% and 50 MPa/350%, respectively [17]. The toughness of TPU in film and fibre form was reported as 111 J g^−1^ [14] and 567 J g^−1^ [15], respectively.

Two- and three-dimensional textile assemblies have been extensively used to reinforce polymer-matrix composites, in numerous applications [18]. However, the study of synthetic nonwoven reinforcements of different structural and compositional formats has been limited. Nonwoven fabrics are assembled from fibres or filaments by web formation and bonding methods. Mechanically bonded nonwovens produced by methods such as needlepunching and hydroentangling rely on fibre entanglement and frictional resistance to slippage for their strength [19]. Compared to preforms with planar fibre orientation, the out-of-plane deflections of fibre segments in mechanically bonded nonwovens can impart mechanical properties in three dimensions [20]. The absence of chemical binders or fibre fusion within the fabric structure provides for high porosity as well as a large accessible fibre surface area for matrix-fibre adhesion. Additionally, nonwoven fabric production involves fewer process steps than conventional weaving or similar textile formation processes and the production is inherently faster in terms of linear output [19] and can offer relatively low cost reinforcement, compared to conventional textile preforms. Nonwoven fabrics have a high thickness to weight ratio, as dense fibre packing is difficult to achieve at weight fractions equivalent to woven, knitted and braided fabrics. High solid volume fractions are therefore challenging to produce in nonwoven reinforced composites, owing to lack of fibre alignment, but the ability to modulate fibre orientation offers tailoring of properties in different directions [21]. The modulus of polyurethane matrix composites is known to increase with higher fibre volume fractions. Epstein and Shishoo [22] used nonwovens to reinforce elastomeric matrix in structural reaction injection moulding (SRIM) and found a decrease in the resin flow as the volume fraction of the nonwoven reinforcement increased, and resin flow was also found to be affected by fibre orientation. Acar and Harper [23] employed hydroentangled nonwoven preforms, composed of viscose, polyester and blend of polyester/viscose/polypropylene, in polyester resin. The mechanical properties of the nonwoven fabrics and composites were compared at different fibre volume fractions, and while improvements in tensile strength were limited but good energy absorbing properties of the reinforced composites were reported. Wang [21] compared nonwoven glass fibre reinforcements to that of woven reinforcements and concluded that a nonwoven can be an effective reinforcement for flat panels and moulded parts with single curvature.

The correct selection of fibre chemical composition, fibre orientation, fibre dimensions and fibre surface properties is known to be critical in terms of influencing the reinforcement behaviour of an elastomeric composite [24,25]. The tensile strength and modulus of fibres depends on interatomic and intermolecular bonds. Consequently, reinforcing behaviour can be modulated by changing the type of fibre in the TPU matrix composite [26]. High modulus fibres are routinely selected for demanding composite applications, but elsewhere when the functional requirements are less demanding, standard textile fibres have potential to facilitate lower cost composite production, or weight-saving whilst still meeting target performance specifications.

Polyethylene, polypropylene, polyester, polyamide and regenerated cellulose (viscose, lyocell) are the most commonly employed polymers for man-made fibre production, with polypropylene, polyester and viscose being particularly important for nonwoven applications [19]. Clearly, the selection of fibre type is also limited by the thermal properties of the matrix material and processing conditions in composite manufacturing. Owing to these limitations, polymers with relatively low melting points such as polyethylene, polypropylene were unsuitable for the present study. Hence fibres thermally stable at temperatures higher than 220 °C were selected: polyamide 6,6 (T_m_ 240–265 °C), polyethylene terephthalate (PET, T_m_ 245–265 °C), Lyocell (starts to degrade above 300 °C) and polyphenylene terephthalamide (PPTA, starts to degrade above 400 °C). PPTA (para-aramid) fibre is commonly employed for high performance applications and is marketed as Kevlar^®^ (Dupont, Wilmington, DE, USA) and Twaron (Teijin, Osaka, Japan). The selected fibres also offer a range of moduli with values for PPTA, lyocell, PET and PA6 being 58–95, 22–31 [27], 6–11, 3.0–6.5 GPa, respectively [28]. Whilst both consist of regenerated cellulose, the modulus of lyocell fibre is at least double that of viscose [27].

Following preliminary results [29], the purpose of the present work was to understand the degree to which nonwoven reinforcements containing standard textile fibres can deliver satisfactory mechanical performance. The mechanical properties of nonwoven preform reinforced flexible TPU composites were compared with those reinforced with polyphenylene terephthalamide (aramid). Additionally, the role of fibre dimensions and the degree of fibre entanglement in the needlepunched preform on prepared composites were investigated.

## 2. Materials and Methods

Lyocell (1.7 dtex, 38 mm), polyphenylene terephthalamide (PPTA) (1.7 dtex, 58 mm), polyethlyene terephthalate (PET) (1.6 dtex, 38 mm, 6.7 dtex, 60 mm and 16.7 dtex hollow fibre, 64 mm respectively), and polyamide 6,6 (3.3 dtex, 50 mm) fibres were carded (nominal mass area density of 70 g m^−2^, parallel-laid) and pre-needled (42 punches cm^−2^) to form nonwoven preforms. Thermoplastic polyurethane (TPU, Elastollan^®^ A C 88 A 12, Shore hardness 88 A, BASF Polyurethanes GmbH) was pressed in to flat sheets (≈315 g m^−2^) and employed as a matrix material. Elastollan^®^ A C 88 A is a polyester based TPU and exhibits good tensile strength and excellent tear strength properties. The respective nonwoven webs were sandwiched between two layers of TPU sheet and compression moulded at 200 °C and 20 kg cm^−2^ (1.96 MPa) to achieve a nominal fibre content of 10 wt % in a composite of dimensions 15 × 20 cm^2^. The fibre volume fraction was calculated by [30]. The mean fibre volume fraction was 9% ± 1%. The estimated volume fractions for the lyocell, PPTA, PET and PA nonwoven reinforced TPU composites were 8.1%, 8.5%, 8.8% and 10.5% respectively.

(1) $$V_{f}=\frac{1}{\left(1\;+\;\frac{\rho_{f}}{\rho_{r}}\left(\frac{1}{w_{f}}\;-1\right)\right)},$$

where, *ρ_f_* = density of fibre; *ρ_r_* = density of resin; *w_f_* = fibre weight fraction.

To determine fibre mechanical properties, single fibres were mounted on card frames, conditioned for 24 h, at 20 °C and 65% relative humidity, and tensile strength was evaluated on a universal testing machine (5540, Instron, Norwood, MA, USA) with pneumatic fibre grips and 5 N load cell, according to ASTM D3822-14 [31] (20 mm gauge length, Constant rate of extension, CRE: 12 mm min^−1^). The nonwoven webs (NWSP 110.4-15 [32], 100 mm gauge length and 50 mm wide, CRE: 100 mm min^−1^) and dog-bone shaped tensile bars from nonwoven-reinforced composites (ISO 527-4:1997 [33], 55 mm gauge length and 10 mm wide, CRE: 10 mm min^−1^) were tested to determine tensile strength on a universal testing machine (Z010, Zwick Roell, Kennesaw, GA, USA) with mechanical jaws. The reported stress is actually engineering stress based on in the initial cross-sectional area of the composite. For scanning electron microscope (SEM) characterisation, the prepared composites were frozen to −20 °C, cross-sectioned, and cut with a fresh razor blade. The samples were sputter coated with gold and imaged on a tungsten source scanning electron microscope S-2600N, Hitachi, Tokyo, Japan). To study the effect of needlepunching density on the mechanical properties of the preforms and resulting composites, PET staple fibres (1.6 dtex, 38 mm) were parallel-laid to produce a nominal mass area density of 70 g m^−2^ webs. The prepared webs were bonded with three different needlepunching densities (42, 84 and 168 punches cm^−2^). The tensile strength of the preforms and composites were carried out according to aforementioned methods.

## 3. Results and Discussion

In line with aims of the study, this section is sub-divided to consider the effect of fibre type, fibre linear density and needlepunching density on the mechanical properties of the nonwoven reinforced elastomeric composites.

### 3.1. Effect of Fibre Type and Linear Density

**Figure 1:**
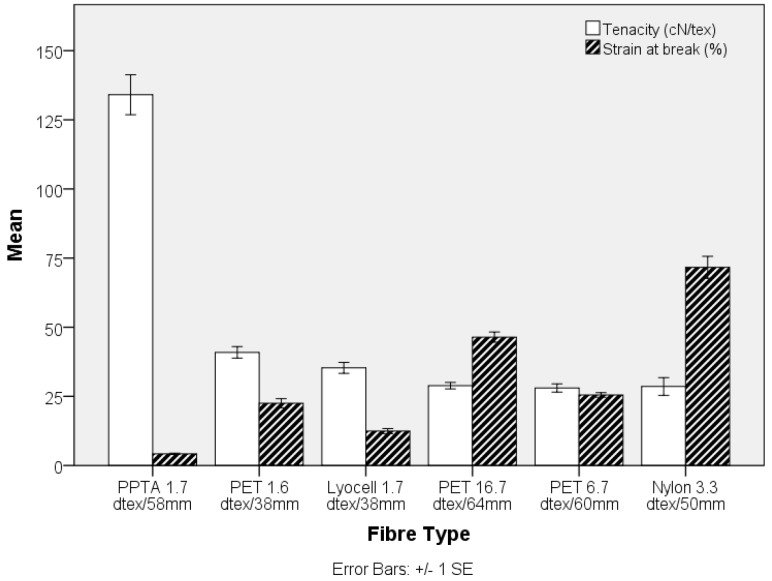
Tensile strength of single fibres.

**Figure 2:**
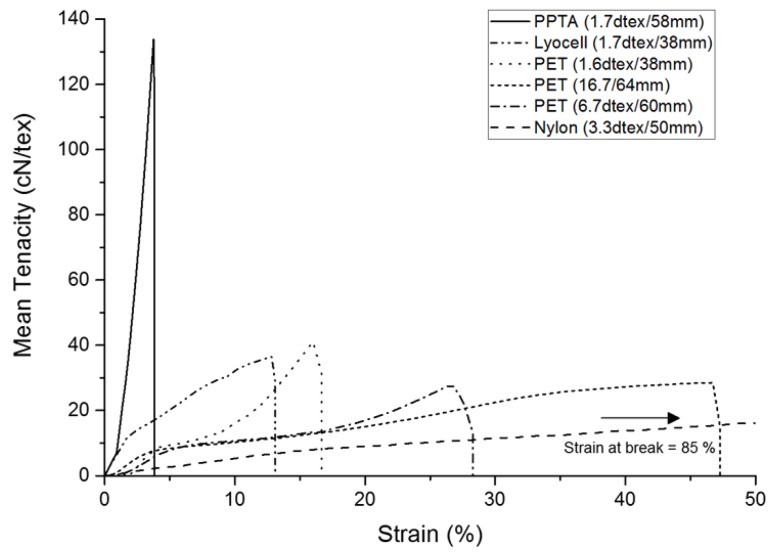
Representative tenacity-strain curves of single fibre tensile testing in Figure 1.

Fibre tensile properties, length and fibre-matrix adhesion are key parameters affecting the final mechanical behaviour of the reinforced composites. The critical fibre length for matrix-to-fibre load transfer depends on the fibre diameter, tensile strength and fibre to matrix bond strength. The fibre lengths selected in the current study were substantially higher than the critical fibre length, as reflected by the resulting mechanical properties [28,34]. The fibre-matrix adhesion can be controlled by altering the surface energy of the fibres and/or matrix resin. The effect of fibre (including PET and PPTA) surface treatments on mechanical properties of nonwoven reinforced polyurethane composites has been previously reported [35]. The mean single fibre strength values for each fibre type are given in Figure 1 and representative tenacity-strain curves are shown in Figure 2. The fibres show a range of tensile strength and strain % values, with PPTA exhibiting premium mechanical properties compared to the rest of the fibres. The reported moduli of PPTA, Lyocell, PET and PA6 are 58–95, 22–31[27], 6–11, 3.0–6.5 GPa, respectively [28], which is consistent with the tensile data in Figure 2.

**Figure 3:**
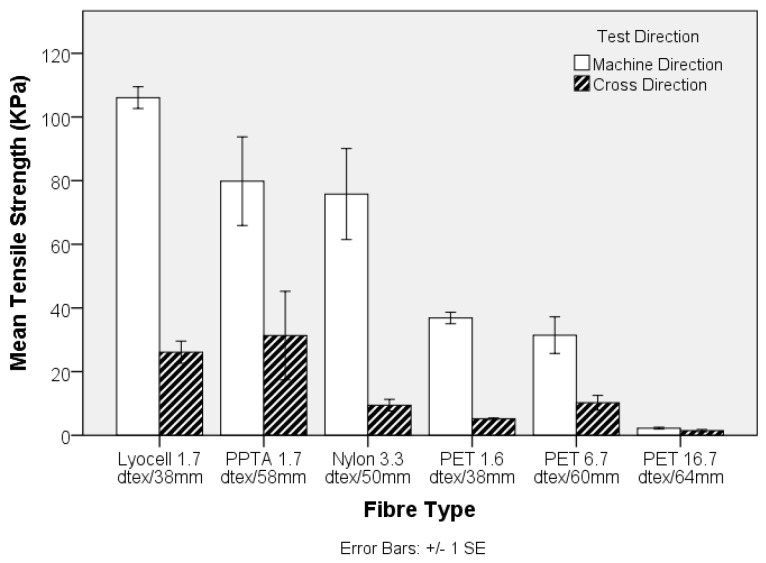
Tensile strength of pre-needled nonwoven preforms in machine- and cross-direction. (Typical stress-strain curve for each sample is given in Figure S1 of Supplementary data).

**Figure 4:**
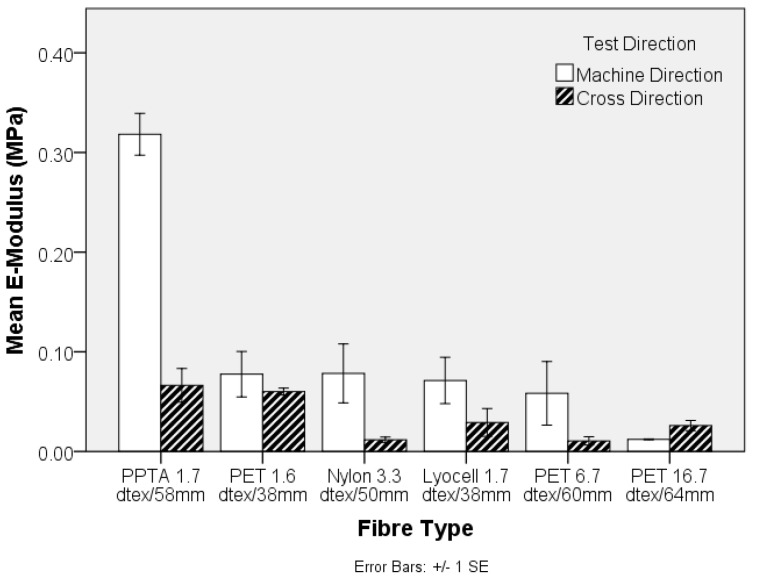
Tensile modulus of pre-needled nonwoven-reinforcements in machine- and cross-direction.

The engineering stress and modulus of the pre-needled nonwoven preform, both in the machine- (MD) and cross-direction (CD), is shown in Figure 3 and Figure 4, respectively. The apparent anisotropy in the preforms, reflected by the MD and CD values is associated with directional variation in the fibre segment orientation, which largely depends upon the method of web formation [36]. In the present study, the nonwoven preforms were based on parallel-laid webs with preferential fibre orientation in the MD. Comparing the tensile strength data in Figure 1 and Figure 3, it is evident that the trend in fibre tensile strength does not correspond to the nonwoven preform tensile strength. At a fixed needle type and needling density, in this instance of 42 punches cm^−2^, development of fibre entanglement and the associated increase in network strength depends on the modulus, inter-fibre friction and bending rigidity of the fibres. The oscillation of the barbed needles during needlepunching induces fibre entanglement and increased structural integrity in the preform. The two contributing effects relate to the capstan effect and the fibre-fibre contact pressures. The capstan effect at fibre crossovers and the contact pressure are the result of inter-looping of fibre segments. This restricts the displacement of fibres, increasing resistance to slippage [37]. Deflection of fibre segments occurs as needle barbs carry the fibre segments and their ability to deflect will depend upon the fibre modulus. For a fibre of circular cross section, the bending stiffness is linearly proportional to the Young’s modulus of the fibre, is affected by fibre length and increases as a function of the fourth power of fibre diameter [38,39]. Therefore, the lyocell fibres produced the strongest nonwoven preforms (Figure 2) despite the fact that PPTA fibre exhibited the highest strength and modulus (Figure 1 and Figure 3). Furthermore, pre-needled PET preforms containing a range of fibre linear densities exhibited inferior mechanical properties, especially coarse (16.7 dtex) PET fibres did not markedly influence results. The 16.7 dtex fibres were the coarsest among all fibres such that fewer fibre segments could be carried by the needle barbs to effect entanglement of the web. The tensile modulus (Figure 4) of the nonwoven preforms exhibited high variation and one-way ANOVA and post-hoc analysis revealed that only the PPTA preform were significantly (*p*-value 0.000) different, in the MD, from the rest of the samples.

**Figure 5:**
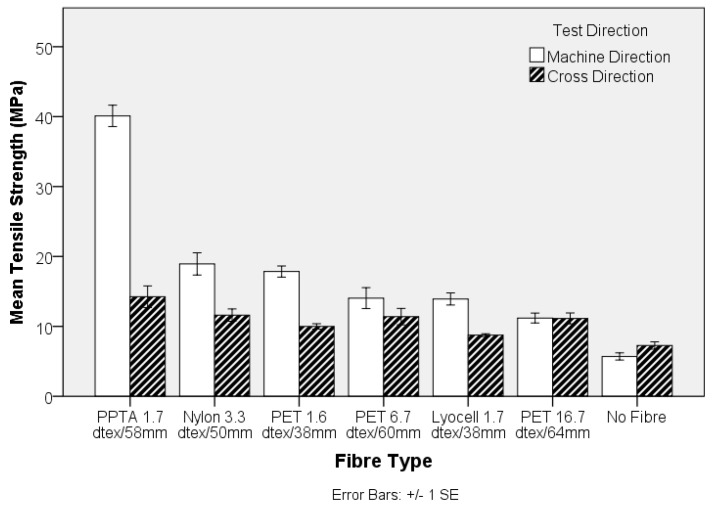
Tensile strength of nonwoven-reinforced thermoplastic polyurethane (TPU) composites.

**Figure 6:**
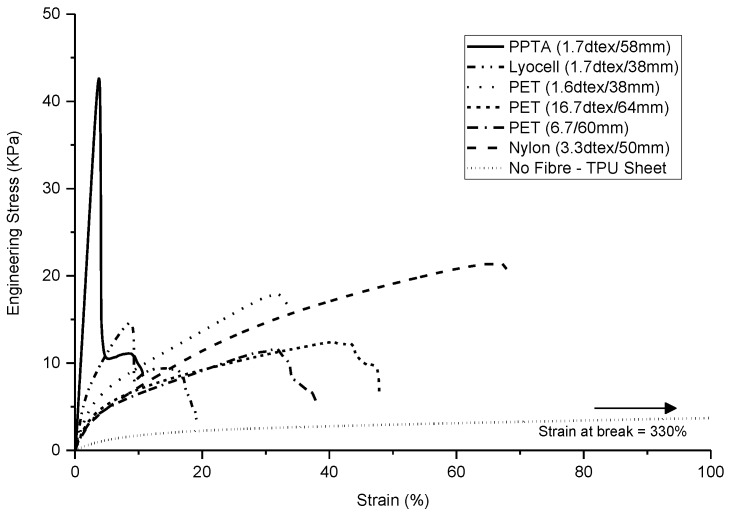
Representative stress-strain curves of nonwoven-reinforced thermoplastic polyurethane (TPU) composites reported in Figure 5. (The graph is limited to 100% strain for clarity. The non-reinforced TPU sample exhibits maximum stress of 5.7 KPa and strain at break of 330%).

The maximum stress of the nonwoven-reinforced TPU composites in the MD and CD is shown in Figure 5. A critical volume fraction is required before fibres contribute to composite strength [40], and in the present study, with a low solid volume fraction of 9% ± 1%, inclusion of a nonwoven preform was found to improve the tensile strength and modulus of the composites compared to that of the unreinforced TPU sheet. Previously, the composite strength has been linked to nonwoven preform strength [23]. In the current study, the composite strength was strongly related to the mechanical properties of the reinforcing fibre, consistent with the findings of Epstein and Shishoo who reported that the modulus of a composite is influenced by the single fibre modulus [35]. This is further evidenced by the stress-strain responses in Figure 6. The tensile strength and modulus of the TPU sheet substantially increases as a result of fibre reinforcement, according the intrinsic fibre properties. Two important considerations are the fibre mechanical properties as well as the matrix-fibre adhesion. Among all samples, the PPTA and lyocell fibre nonwoven reinforced samples exhibited a double peak in the stress-strain curve, where the first peak may be associated with de-bonding of the fibre and matrix phase [41]. The de-bonding is likely to be associated with the low strain % at break of the PPTA and lyocell fibres. Previously, it has been reported that a typical load-elongation curve of the hydroentangled (PET) nonwoven reinforced composite exhibits many peaks and troughs, linked to local failure of matrix and fibres, at a comparable fibre volume fraction of 11% [23]. However, in the current study, the stress-strain curves were smooth.

**Figure 7:**
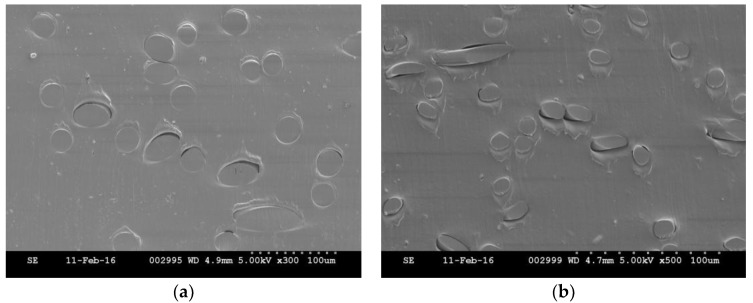
Scanning electron microscope (SEM) images of nonwoven-reinforced TPU composite cross-sections in the machine direction: Polyethylene terephthalate fibres (6.7 dtex) (**a**) and lyocell fibres (**b**).

The high viscosity of elastomeric matrix can hinder resin infiltration particularly as the fibre volume fraction increases [22,42]. Furthermore, void formation during manufacture is a known problem affecting the mechanical properties of composites [34]. Freedom from void formation was confirmed by assessment of SEM images, Figure 7. The high number of circular fibre cross-sections in the SEM images confirms the preferential fibre orientations in the MD. The magnitude of uniaxial nonwoven preform strength depends on the fibre segment orientation distribution and fibre entanglement. For nonwoven reinforced composites, fibre orientation plays the major role as the fibres are bonded in the matrix phase. This is reflected by the lack of correlation between the preform (Figure 3) and composite’s maximum engineering stress (Figure 6). The directional geometrical arrangement of fibres can therefore be tailored to modulate composite properties, by process-structuring the nonwoven preform during its manufacture.

**Figure 8:**
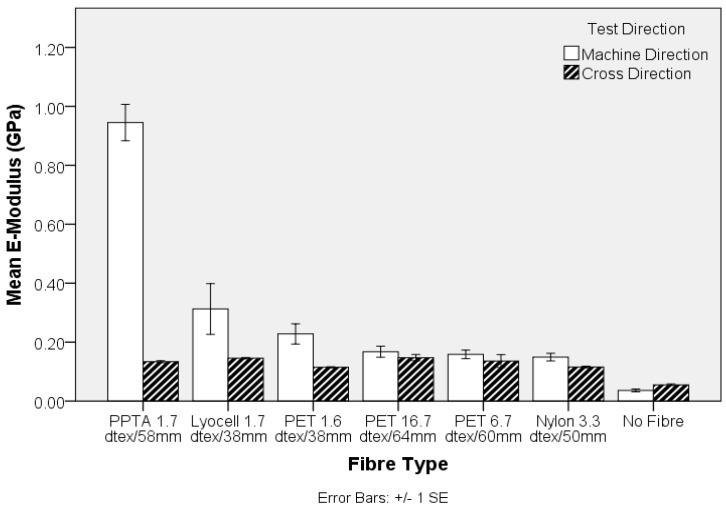
Tensile modulus of nonwoven-reinforced TPU composites.

The inclusion of the nonwoven reinforcement, at a mean solid volume fraction of 9% ± 1%, in a TPU matrix results in an expected increase in Young’s Modulus of the composites (Figure 8). With reference to the values obtained for PPTA, the crucial influence of fibre modulus on the corresponding composite values are highlighted in Figure 8. Despite the fibre modulus being substantially lower than PPTA, reinforcement with standard textile fibres at a solid volume fraction of only 9% ± 1% is capable of more than doubling the modulus of the composite, depending on the selected fibre type, (Figure 8).

**Figure 9:**
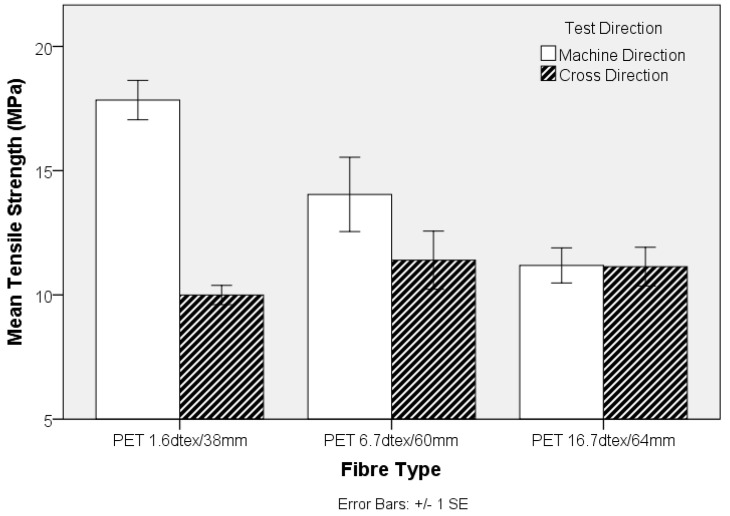
Tensile strength of three polyethlyene terephthalate (PET) nonwoven-reinforced TPU composites (extracted from Figure 5).

**Figure 10:**
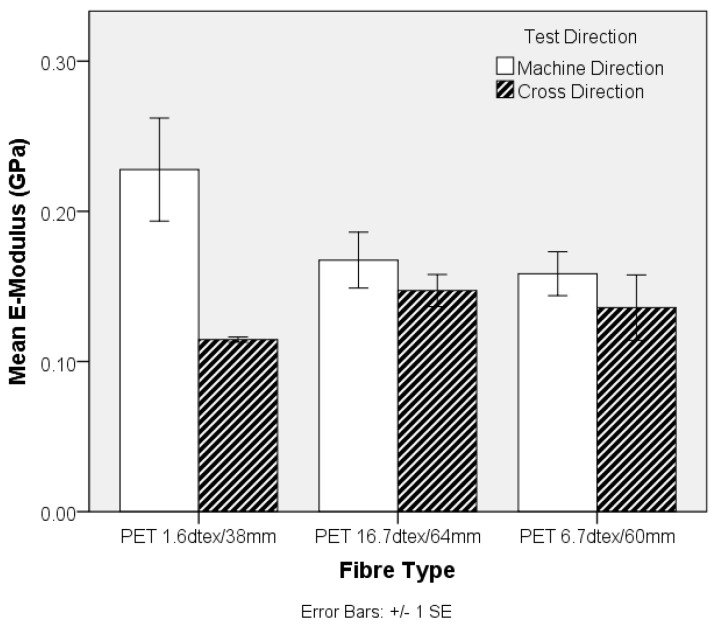
Tensile modulus of the PET nonwoven-reinforced TPU composites (extracted from Figure 8).

The influence of fibre diameter on the nonwoven preform was explored by examining the properties of TPU composites produced from three different PET linear densities. The MD tensile strength shown in Figure 9 decreases with increasing fibre linear density, whereas there is no clear trend in the CD. Clearly, the higher specific surface area resulting from an increase in fibre fineness, is likely to increase the total interface surface for matrix to fibre adhesion aiding stress transfer. Similarly, the stiffness of the TPU composite decreases as fibre fineness increases in the nonwoven preform, Figure 10. Furthermore, the strength of the composite samples containing fibres of different linear density can be related to differences in single fibre mechanical properties, Figure 1.

### 3.2. Effect of Needlepunching Density

**Figure 11:**
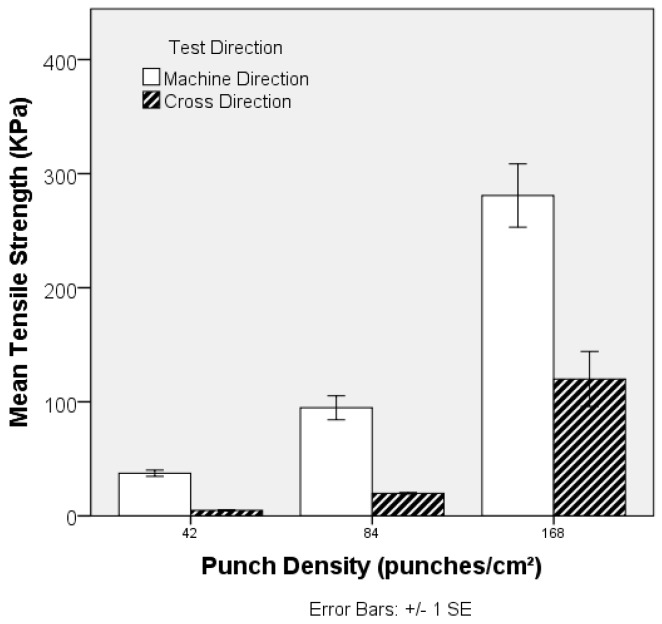
Effect of punch density on tensile strength of PET (1.6 dtex, 38 mm) nonwoven preforms. (Typical stress-strain curve for each sample is given in Figure S2 of Supplementary data).

**Figure 12:**
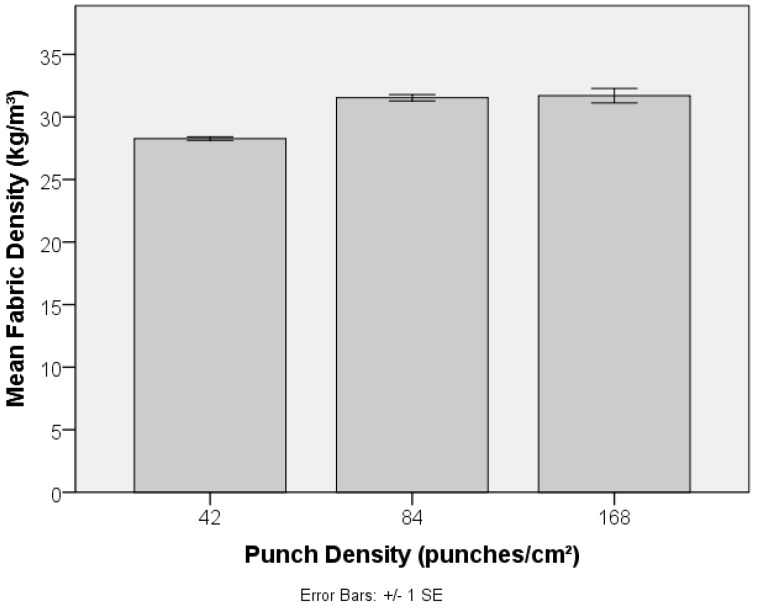
Effect of punch density on density of PET (1.6 dtex, 38 mm) nonwoven preforms (fabrics).

**Figure 13:**
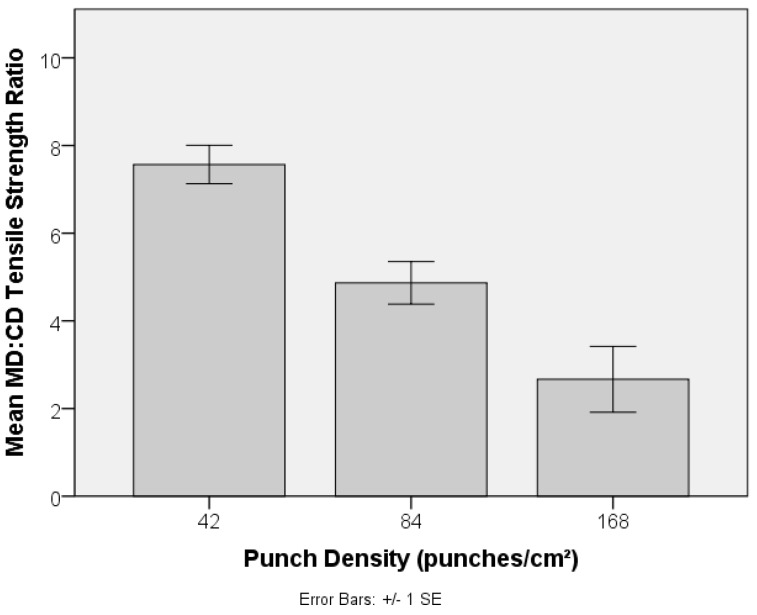
Effect of punch density on (MD:CD) tensile strength ratio of PET (1.6 dtex, 38 mm) nonwoven preforms.

**Figure 14:**
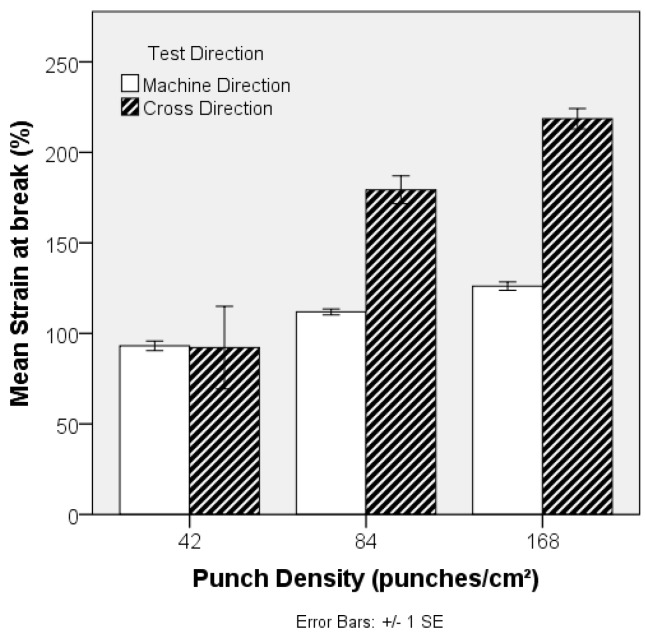
Effect of punch density on strain (%) of PET (1.6 dtex, 38 mm) nonwoven preform.

Local fibre arrangement and fibre entanglement in the preform (PET-1.6 dtex, 38 mm) is adjusted by increasing the punch density during its manufacture, and the impact on its resulting strength is revealed in Figure 11. Needlepunching of staple fibre webs initially increases consolidation and the degree of fibre entanglement, increasing tensile strength and modulus, until a threshold is reached beyond which out-of-plane fibre segments reduce planar strength and fibre mobility. Excessive needling can also cause fibre breakage and holes on the preform surface [43]. Given the relatively low needling density, such a threshold was not reached in the current study, as is evident by the systematic increase in tensile strength with increasing punch density from 42 to 168 punches cm^−2^. Note that this was not accompanied by a large increase in preform density (Figure 12). Fibres in parallel-laid nonwovens are preferentially oriented in the MD and the observed decrease in anisotropy, reflected by the MD:CD strength ratio in Figure 13, can be associated with local in-plane re-orientation and out-of-plane fibre reorientation of fibre segments as the punch density increases. The strain at break %, Figure 14, increased with increasing punch density. The higher level of entanglement may increase the normal force on the existing fibre to fibre cross-over points facilitating higher elongation of the fibre segments between bond points, and increased tensile strength.

**Figure 15:**
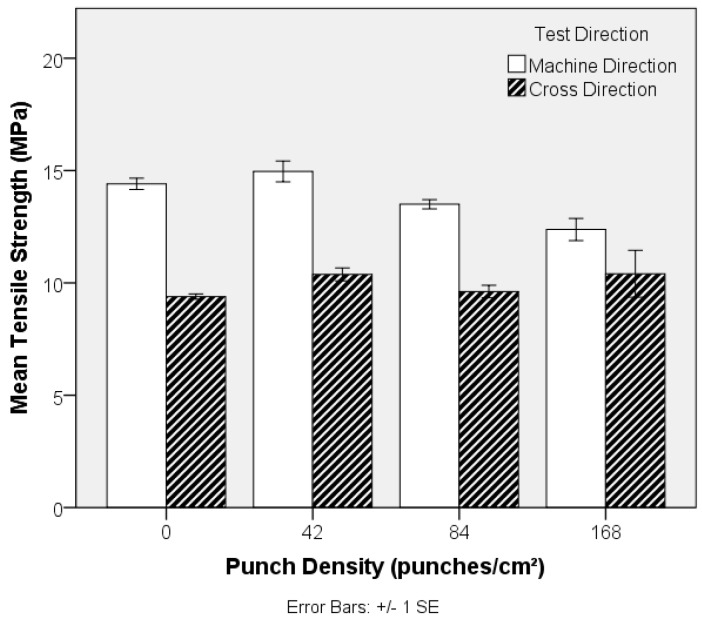
Effect of punch density on tensile strength of PET nonwoven (1.6 dtex/38 mm) reinforced flexible composites. (Typical stress-strain curve for each sample is given in Figure S3 of Supplementary data).

While increasing the punch density increases the tensile strength of the preform (Figure 11), there is a small but significant decrease in the tensile strength of the TPU composite, Figure 15. One-way ANOVA analysis shows a significant (*p*-value 0.006) effect of different levels of punch density on tensile strength in the MD and no significant effect in the CD (*p*-value 0.0604). Further post-hoc analysis in the MD reveals that only 168 punches cm^−2^ is significantly different from samples at 0 (*p*-value 0.021) and 42 (*p*-value 0.006) punches cm^−2^. The small decrease in tensile stress at the highest punch densities may be attributed to out-of-plane fibre segment deflections and a slight increase in the preform (fabric) density, which may be expected to increase the resistance to TPU flow infiltration during composite manufacture due to a decrease in the intrinsic permeability.

## 4. Conclusions

The mechanical properties of single fibres, nonwovens and reinforced composites were evaluated and the key findings can be summarised as follows:

- Conventional textile fibres such as PET and lyocell have the potential to increase, six and nine times respectively, the tensile modulus of compression moulded TPU composites when introduced as nonwoven preforms with solid volume fractions of <10%. In addition, reinforcement of TPU moulded composites with PET- and lyocell nonwovens led to a minimum 2.5 fold increase in tensile strength compared to unreinforced TPU. This is substantially lower than can be achieved with comparable nonwoven PPTA fibre preforms, but there may be cost benefits in selecting lower cost reinforcements for less demanding applications.
- High strength fibres do not essentially produce high strength nonwoven preforms, with all other parameters constant. The tensile properties of nonwoven-reinforced composites are mainly dependent upon fibre tensile properties and fibre segment orientation distribution. Though the tensile strength of the nonwoven preform does not markedly contribute to the tensile strength of the composite, the structural and dimensional modifications associated with increased levels of bonding such as greater fibre entanglement, fibre re-orientation, fabric density and fibre volume fractions can influence resulting mechanical properties.
- Cross-sectional analysis of nonwoven-reinforced composite parts, revealed void-free embedding of conventional fibres in the TPU matrix.
- Modulating the degree of fibre entanglement and mechanical bonding in the nonwoven preform by adjusting the level of needling resulted in a small but significant decrease in tensile strength of the TPU composite. Based on a fixed fibre type and solid volume fraction, increasing fibre linear density reduced the strength of the nonwoven-reinforced TPU composite.

## Supplementary Materials

The following are available online at http://www.mdpi.com/1996-1944/10/6/618/s1, Figure S1: Typical stress-strain curves for tensile strength of pre-needled nonwoven preforms in machine- and cross-direction (reference to Figure 3 of the main document), Figure S2: Typical stress-strain curves for effect of punch density on tensile strength of PET (1.6dtex, 38mm) nonwoven preforms (reference to Figure 11 of the main document), Figure S3: Typical stress-strain curves for effect of punch density on tensile strength of PET nonwoven (1.6dtex/38mm) reinforced flexible composites (reference to Figure 15 of the main document). Typical stress-strain curves for Figure 3, Figure 11 and Figure 15 are provided.
