# Supplementary material for: Mechanical Properties of Nonwoven Reinforced Thermoplastic Polyurethane Composites
Source: Materials (Basel). 2017 Jun 5;10(6):618. doi: 10.3390/ma10060618 (PMC5553526; doi:10.3390/ma10060618)
Supplement: Supplementary file 1 [file materials-10-00618-s001.pdf]

materials

Supplementary Material

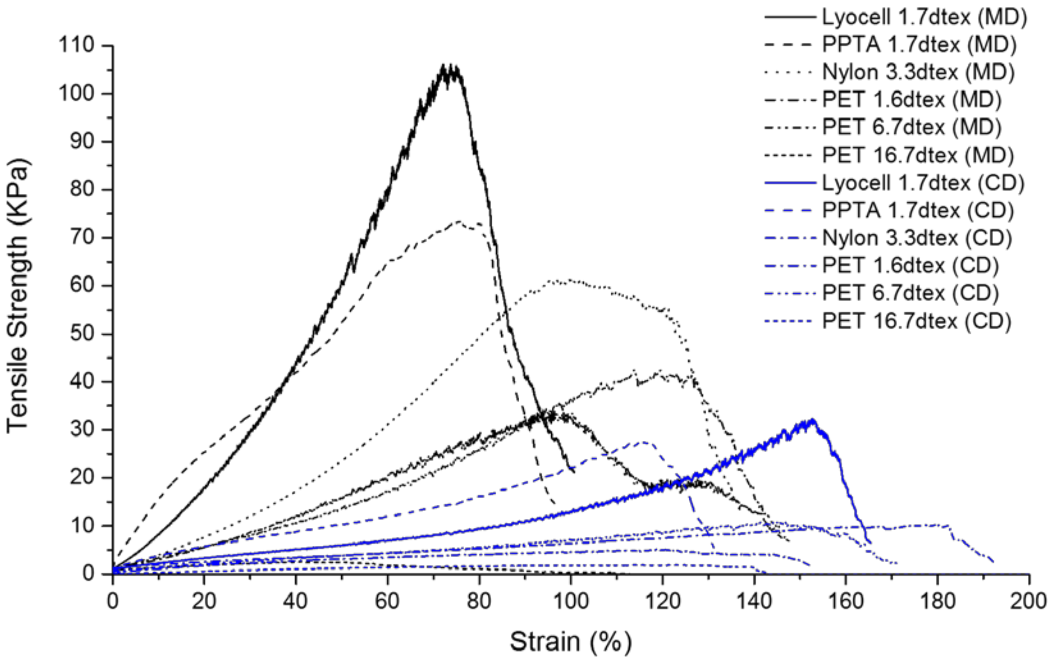

Figure S1. Typical stress-strain curves for tensile strength of pre-needled nonwoven preforms in machine- and cross-direction (reference to Figure 3 of the main document).

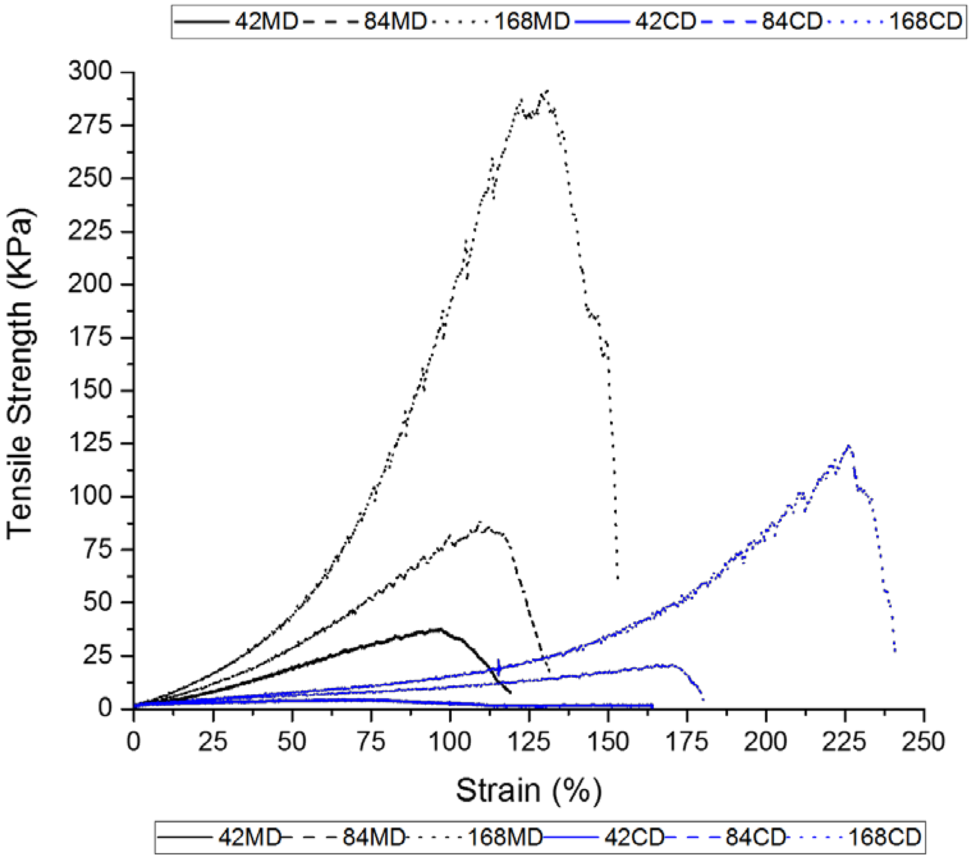

Figure S2. Typical stress-strain curves for effect of punch density on tensile strength of PET (1.6dtex, 38mm) nonwoven preforms (reference to Figure 11 of the main document).

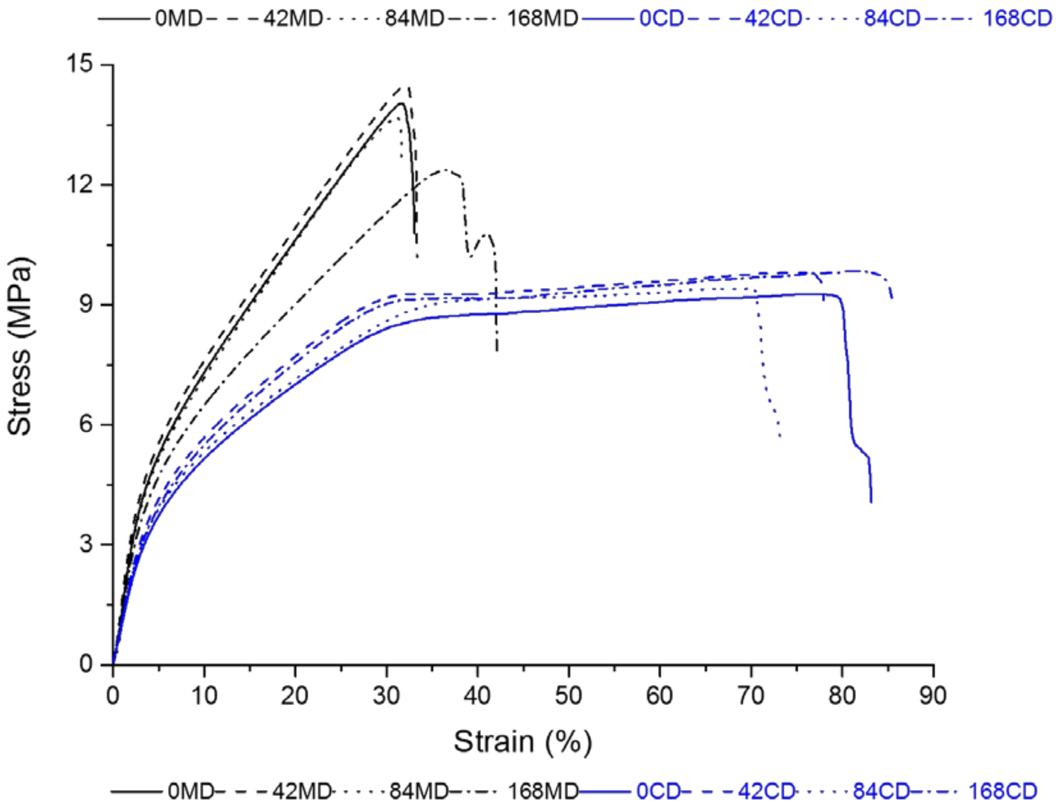

Figure S3. Typical stress-strain curves for effect of punch density on tensile strength of PET nonwoven (1.6dtex/38mm) reinforced flexible composites (reference to Figure 15 of the main document).
